# Growth Performance, Biochemical Composition and Nutrient Recovery Ability of Twelve Microalgae Consortia Isolated from Various Local Organic Wastes Grown on Nano-Filtered Pig Slurry

**DOI:** 10.3390/molecules27020422

**Published:** 2022-01-10

**Authors:** Min Su, Marta Dell’Orto, Barbara Scaglia, Giuliana D’Imporzano, Alessia Bani, Fabrizio Adani

**Affiliations:** 1 Gruppo Ricicla Lab., Dipartimento di Scienze Agrarie e Ambientali—Produzione, Territorio, Agroenergia (DiSAA), Università degli studi di Milano, Via Celoria 2, 20133 Milan, Italy; min.su@unimi.it (M.S.); marta.dellorto@unimi.it (M.D.); barbara.scaglia@unimi.it (B.S.); giuliana.dimporzano@unimi.it (G.D.); 2 School of Life Sciences, University of Essex, Wivenhoe Park, Colchester CO4 3SQ, UK; alessia.bani90@gmail.com

**Keywords:** amino acid, biochemical compositions, fatty acid, microalgae-microbial consortia, nutrient recovery, organic waste

## Abstract

This paper demonstrated the growth ability of twelve algae-microbial consortia (AC) isolated from organic wastes when a pig slurry-derived wastewater (NFP) was used as growth substrate in autotrophic cultivation. Nutrient recovery, biochemical composition, fatty acid and amino acid profiles of algae consortia were evaluated and compared. Three algae-microbial consortia, i.e., a *Chlorella*-dominated consortium (AC_1), a *Tetradesmus* and *Synechocystis* co-dominated consortium (AC_10), and a *Chlorella* and *Tetradesmus* co-dominated consortium (AC_12) were found to have the best growth rates (µ of 0.55 ± 0.04, 0.52 ± 0.06, and 0.58 ± 0.03 d^−1^, respectively), which made them good candidates for further applications. The ACs showed high carbohydrates and lipid contents but low contents of both proteins and essential amino acids, probably because of the low N concentration of NFP. AC_1 and AC_12 showed optimal ω6:ω3 ratios of 3.1 and 3.6, which make them interesting from a nutritional point of view.

## 1. Introduction

Microalgae cultivation is considered to be an efficient tool in the framework of a circular economy, combining wastewater treatment and the production of valuable biomass for various purposes [1]. Microalgae cultivation implies high consumption of water, inorganic nutrients and CO_2_ [2]. The utilization of chemical fertilizers as nutrient sources is costly and reduces the environmental sustainability of microalgae-based processes [3]. The chemical compositions of several kinds of wastewater are quite similar to the culture media usually adopted for microalgae growth; hence, the possibility to recover inorganic nutrients, water and CO_2_ from wastewaters and organic wastes provides an environmentally friendly and cheap method for algae production [2]. In particular, inorganic nitrogen (N) and phosphorus (P) uptake by microalgae can be used as an efficient bioremediation tool for wastewater treatment, transforming these nutrients into energy-rich biomass, which can be further processed to make biofuels or other valuable products such as biofertilizers, bioplastics and so forth [4].

One of the main bottlenecks hampering the full exploitation of this tool is that among the wide variety of microalgal species present in nature, only a few species are currently known to survive and grow in wastewater or highly carbon-rich wastes [1]. It has been reported that compared to algae monospecies cultures, algal-bacteria consortia may be more suitable for cultivation on wastewater by acting symbiotically, especially in terms of preventing contamination and enabling long-term cultivation in open systems [2]. In this mutualistic equilibrium, the O_2_ released by algal photosynthesis is utilized by aerobic-heterotrophic bacteria to mineralize organic compounds, and bacterial respiration provides CO_2_ as a carbon (C) source to the algae. Additionally, it has been demonstrated that algae-bacteria consortia systems take up nutrients from digestate more efficiently than monoculture systems [5].

Several types of organic wastes and wastewaters are characterized by the presence of ammonium (NH_4_^+^/NH_3_) as a proportion of the total N content or as the unique N form. Ammonium is the preferred N source for microalgae since, unlike nitrate, it does not need energy-consuming reduction steps once it is taken up by the cells [6]. However, the ammoniacal N form can also be toxic for microalgae, its toxicity depending on the equilibrium between the ionized (NH_4_^+^) and unionized (NH_3_) water-soluble forms, and volatile (NH_3_) forms, which is in turn determined mainly by pH and secondarily by temperature [7]. Although both N forms are toxic to some extent, NH_3_ is the more toxic since it is uncharged and therefore able to cross cell membranes in an uncontrolled way, impairing the pH gradient across thylakoid membranes (uncoupling photophosphorylation) and interfering with PSII, affecting photosynthesis [6]. On the other hand, NH_3_ is very volatile and can be lost to the atmosphere, especially in aerated culture systems [7]. Therefore, the feasibility of using NH_4_-containing wastewaters as the nutrient source for microalgae culture depends mainly on the possibility of matching the wastewater total NH_4_-N concentration and the tolerance of the cultured microalgae. Collos and Harrison [7] detected a wide variability among microalgae phyla in the optimal, tolerated and toxic concentrations of ammonium, providing evidence for a potentially high biodiversity that can be exploited to optimize the use of microalgae for N recovery from wastewater.

This work falls within a broad project funded by the Lombardy Region (North Italy), aimed at both studying and isolating indigenous, autochthonous algae-microbial consortia (ACs) from various organic wastes with different origins, forms and consistencies [8], to be used for technological applications to treat organic wastes, removing nutrients and producing useful biomass both in the lab (this paper) and at pilot scale. Thus, the main objective of this study was to examine and compare the potential of twelve microalgae consortia, originally selected from different waste streams rich in diverse N forms, to be cultivated using a nano-filtered permeate from pig slurry as the growth medium, rich in NH_4_-N. We evaluated the growth performance, nutrient removal efficiency and biomass quality in terms of biochemical composition, along with fatty acid and amino acid profiles, to give insight into the most robust algae consortia, especially in regards to their adaptability to NH_4_-N sources. In a further experimental step, these isolated microalgae consortia were tested in open reactors at a demonstration level (TRL 5–6), aiming to understand how algal communities are influenced by shifting the cultivation modality from closed reactors to open reactors, as well as to evaluate whether the dominant microalgae species from closed reactors could still be dominant in open reactors or whether they could be colonized by other species/bacteria/predators.

## 2. Results and Discussion

### 2.1. Characterization of Nano-Filtered Permeate (NFP)

NFP represented the result of nano-filtration of twice S/L separated pig slurry (see Section 3.1) that, under real conditions (operating full-scale plant), is typically subjected to a reverse osmosis step, producing a concentrated ammonia solution to be used in agriculture as fertilizer, while the clean water is discharged directly to surface-water bodies (see Section 3.1). Instead, in this paper, NFP was directly used as substrate to grow algae in substitution of a commercial substrate (BG-11). NFP appeared suitable for microalgae growth (Table 1) because it contained 136 ± 0 mg L^−1^ of TN, 97% of which was in the ammonium (132 ± 2 mg L^−1^) form (Table 1), readily available to algae and at a concentration avoiding any toxicity [7]. Several references have reported the inhibition of microalgae growth and production [9,10,11] due to high free ammonia concentrations. Free ammonia passes through biological membranes more easily than the ionized NH_4_^+^ form, causing the dissipation of the thylakoid transmembrane proton gradient [11], affecting the Oxygen Evolving Complex (OEC) and, afterward, blocking electron transport in PSII [12,13]. Despite this, several references reported the adaptability of different species to high ammonia concentrations due to the effect of long-term acclimation [14]. Therefore, microalgae consortia isolated from high ammonia-content wastes, such as the ones used in this work, have the potential to cope better with high ammonia concentrations. Additionally, the multistep separation carried out resulted in low turbidity of the NFP, which was optimal for algal growth (Table 1). Lastly, NFP contained many other useful nutrients, of which the concentrations were not far from those reported for BG-11 (Table 1), except for the low TP content (0.5 ± 0 mg L^−1^) that can potentially cause low algae growth capacity. Thus, P was extra-added, giving a final content similar to that of the BG-11 substrate.

### 2.2. Organic Waste Sources of Microalgae Consortia

The algae consortia (AC) were previously isolated from sixteen organic wastes of different types (solid/slurry/liquid fractions), having different origins (cow slurry, urban municipal wastewater, sewage sludge) and sampled from plants/farms located in Northern Italy, around noon in January. The sampling and origin details can be found in Table S1 (Supplementary Materials), where the waste samples were marked as S1 to S16.

The chemical characterization of the sixteen wastes differed greatly in terms of total solids (TS), volatile solids (VS), pH, ammonium, total alkalinity (TA), total volatile fatty acids and macro and microelement compositions [8]. For instance, TS varied from 15 ± 1 g kg^−1^ to 257 ± 10 g kg^−1^ while VS was from 8 ± 0.2 g kg^−1^ to 221 ± 0.6 g kg^−1^. pH ranged between 6.4 ± 0.3 and 9.1 ± 0.2, TA varied widely between 2.1 ± 0.1 g and 18.7 ± 0.5 CaCO_3_ kg^−1^ and ammonium content ranged from 0.3 ± 0 up to 3.5 ± 0.4 g kg^−1^. The broad variety was also found in the macro and microelement compositions [8].

### 2.3. Microalgae Consortia Growth

The algae consortia (AC), previously characterized for the presence of both eukaryotes and prokaryotes (Table 2), can be classified as follows: a *Chlorella*-dominated consortium (AC_1), a *Tetradesmus*-dominated consortium (AC_6), *Chlorella* and *Synechocystis* (Cyanobacteria) co-dominated consortia (AC_2, AC_3, AC_4, AC_5, AC_9 and AC_11), *Tetradesmus* and *Synechocystis* co-dominated consortia (AC_7 and AC_10), a *Chlorella* and *Tetradesmus* co-dominated consortium (AC_12) and, finally, a low algae presence consortium (AC_8). The percentage of compositions was referred to the relative abundances of the operational taxonomic units (OTUs) assigned to each genus with respect to the total OTUs assigned to Eukaryotes or Prokaryotes, respectively.

Previous findings [8] indicated that the metagenomics compositions of the algae consortia directly isolated from organic wastes (OBs) and then cultivated (CBs) under batch conditions were not significantly different from each other.

The specific growth rates (µ) of the twelve ACs were in the range 0.18 ± 0.01 (AC_11) to 0.58 ± 0.06 d^−1^ (AC_12) (Table 2), with an average of 0.34 ± 0.14 d^−1^ (*n* = 12): these values are in line with those reported for autotrophic microalgae, i.e., 0.2-0.7 d^−1^. In particular, growth rates for *Chlorella* sp. and *Tetradesmus* sp. were reported, on average, as 0.45 ± 0.19 d^−1^ (*n* =11) [15,16,17,18,19,20] and 0.27 ± 0.11 d^−1^ (*n* = 10) [20,21,22,23,24], respectively. Taking into consideration the specific growth rates reported in Table 2 and the results of Tukey’s HSD test, the ACs can be classified as follows: AC_1 = AC_10 = AC_12 > AC_2 = AC_3 = AC_4 = AC_5 = AC_6 = AC_7 = AC_8 = AC_9 = AC_11.

The AC_12, which was co-dominated by *Chlorella* (39.6%) and *Tetradesmus* (32.6%), probably gained a significant competitive advantage regarding growth performance, reaching the highest growth rate (0.58 ± 0.03 d^−1^), indicating that the co-culture of *Chlorella* and *Tetradesmus* promoted biomass growth. Algae co-culture has been reported to benefit the culture performance due to the ability of different species to utilize nutrients and light during growth [25]. However, AC_1 and AC_10, which showed similar growth rates to AC_12 (Table 2), did not show the co-dominance of eukaryotic algae. In these cases, it may be that the prokaryotic community provided a positive effect on algae growth [2]. For example, *Synechocystis* co-cultivated in consortia with *Chlorella* sp. could have promoted cell growth [26], explaining the good growth performance of AC_10. 

In conclusion, AC_1, AC_10 and AC_12 were the best performing ACs, representing good candidates for biomass production using NH_4_-rich wastewaters. Moreover, the co-cultivation of multiple microalgae species and/or bacteria provides not only the advantage of promoting and benefitting the growth of each other, but also reduces the frequency and extent of culture crashes caused by viral, bacterial or fungal infections or predation by protozoa, especially in outdoor conditions [27].

### 2.4. Nutrient Mass Balance

In this section, the ability of ACs to remove nutrients from NFP is reported and discussed, considering that nutrient removal is not only important to clean NFP, but that nutrient uptake is also important for algae growth and biomass composition, as will be discussed in the following sections. 

Nutrient availability plays an important role in governing algae-microbial consortia growth and composition. This point is very important in the case of nutrient recovery from waste streams. Nitrogen is crucial for the growth of microorganisms and is one of the main constituents of vital macromolecules, such as amino acids and proteins, pigments (chlorophyll, phycocyanin, etc.), DNA, RNA, etc. The AC cultures showed a notable ability to assimilate nitrogen (Table 3). On average, 61 ± 15% (*n* = 12) of initial N (TN_initial_) was taken up by algal biomass. Furthermore, the three fastest-growing cultures, i.e., AC_1, AC_10 and AC_12 displayed N uptakes of 54 ± 0%, 62 ± 0% and 53 ± 11% TN_initial_, respectively, along with an average of 56 ± 4% TN_initial_ (*n* = 3). These values were higher than those previously reported for microbial communities growing on wastewater, i.e., 44 ± 6% TN_initial_ (*n* = 6) [28,29,30] and those reported for pure *Chlorella*, i.e., 39.7 ± 12.7% TN_initial_ (*n* = 8) [16,31].

The culture of consortia would affect the metabolism of the algae, resulting in different behaviors in nutrient removal [32] and explaining the variability registered (Table 3). 

Phosphorus, although it makes up less than 1% of the biomass, is essential for algal growth, given that it is involved in many cellular processes [33]. The removal of inorganic phosphorus in algal culture results from two phenomena: biological assimilation and chemical precipitation as insoluble phosphate [34]. Phosphorus content in algal biomass was not measured. However, since the pH of the cultures never reached over 8, i.e., the pH value at which the precipitation of phosphate occurs, it is conceivable that phosphates were mostly removed by active uptake rather than being precipitated as insoluble phosphate salts [35]. 

In this context, the P uptake measured for all ACs was very high, i.e., on average 92 ± 4% P_initial_ (*n* = 12), with results being higher than those of the range reported for *Chlorella* (63–75% P_initial_) grown on anaerobic digestate of dairy manure [36], but comparable with data reported for algae growth on wastewater, i.e., 97% P_initial_ [31].

### 2.5. Biochemical Composition of AC Biomasses

Microalgae gained interest because of their capacity to accumulate important amounts of useful biochemical molecules such as proteins, lipids, carbohydrates and pigments, compared to other biomasses [37]. Therefore, biochemical composition detection is important in characterizing ACs growing on NFP.

Average protein content (Table 3) was, on average, 368 ± 90 g kg^−1^ DM (*n* = 12) and the contents for the fastest growing cultures, i.e., AC_1, AC_10 and AC_12, were 257 ± 0, 382 ± 7 and 273 ± 22 g kg^−1^ DM proteins, respectively. These contents were lower than those reported in the literature for *Chlorella* sp., i.e., 440 ± 129 g kg^−1^ DM (*n* = 8) [38,39,40,41,42] and *Tetradesmus* sp., i.e., 522 ± 173 g kg^−1^ DM (*n* = 2), both cultivated in different waste streams [39,43] containing more N than that of the NFP (N of 136 ± 0 mg L^−1^) used in this work, i.e., N 594 ± 453 mg L^−1^ and 314 ± 90 mg L^−1^, respectively. These figures can explain the lower protein content characterizing ACs. The fact that *Chlorella* cultivated on agricultural wastewater containing ammonia as a N source (100–150 mg L^−1^), similar to that of NFP, showed a protein content in line with those of ACs, i.e., 327 ± 150 g kg^−1^ DM [31,44,45] seems to confirm that total N affected total protein content. 

Lipids are one of the major constituents of microalgal biomass, and their content is typically reported to be in the range of 50–500 mg kg^−1^ DM, depending on the species and cultivation conditions [46]. The lipid content found in this work was, on average, 145 ± 33 g kg^−1^ DM (*n* = 12), in line with data reported for *Chlorella* sp., i.e., 144 ± 98 g kg^−1^ DM (*n* = 9) [38,40,41,47,48], and the data reported for *Tetradesmus* sp., i.e., 216 ± 47 g kg^−1^ DM (*n* = 4) [22,41,47] (ANOVA, *p* < 0.05). The lipid content was, on average, higher than that reported for cyanobacteria, i.e., 99 ± 56 g kg^−1^ DM [48], indicating that the mixed culture (e.g., AC_10) probably led to lower lipid content than the monospecies culture (Table 3). It is worth mentioning that between the three fastest-growing ACs (Table 1), AC_12 achieved relatively high lipid contents, i.e., 230 ± 15 g kg^−1^ DM; therefore, the algae co-culture was probably positive for lipid content (Table 3).

Carbohydrate content in algal cells ranged from 40 to 640 g kg^−1^ DM depending on different algae species and cultivation conditions [49]. In this work, the carbohydrate content in the ACs presented an average of 445 ± 95 g kg^−1^ DM (*n* = 12) (Table 3), much higher than data reported for *Chlorella* sp., i.e., 270 ± 84 g kg^−1^ DM (*n* = 9) [38,40,41,47,48] and *Tetradesmus* sp., i.e., 244 ± 37 g kg^−1^ DM (*n* =4) [22,41,47] (ANOVA, *p* < 0.05). The three fastest growing ACs, namely AC_1, AC_10 and AC_12, revealed carbohydrate contents of 596 ± 4, 425 ± 9 and 472 ± 27 g kg^−1^ DM, respectively.

**Figure 1:**
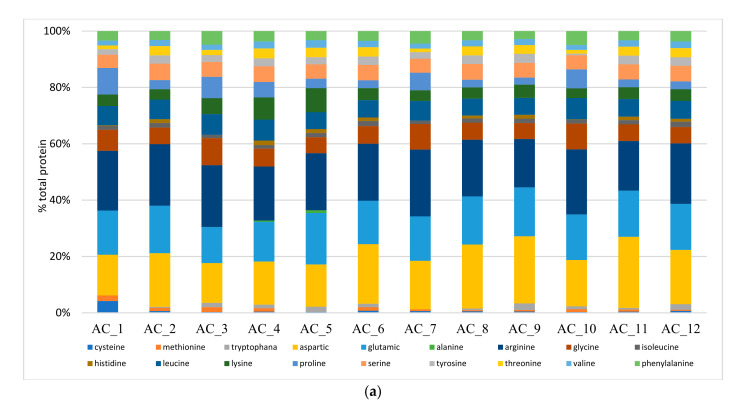

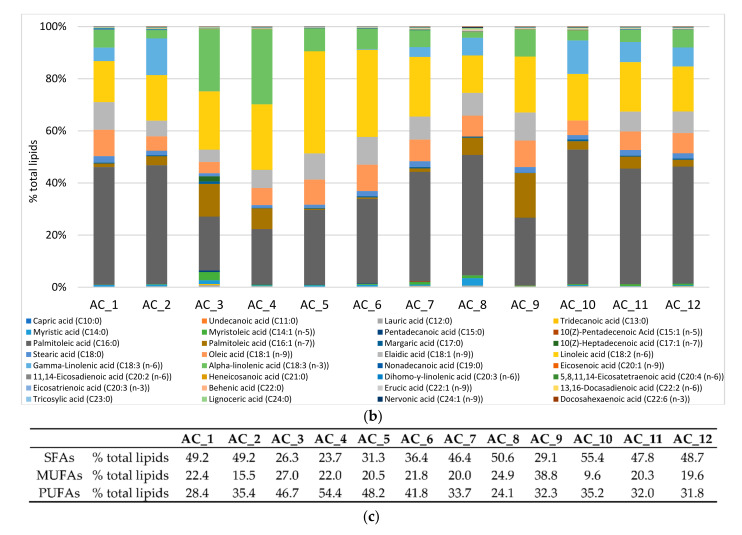
(**a**) Amino acid speciation, (**b**) fatty acid speciation and (**c**) summary of fatty acid compositions.

The data discussed above and those reported in Figure 1 indicate a great variability among ACs for their biochemical composition, depending, above all, on the ACs’ characteristics, since all the other variables affecting biochemical composition would be reduced by the similar growing conditions adopted throughout the experiments [50]. In particular, the results (Table 2 and Table 3) suggested that a higher µ corresponded with higher lipid contents, in agreement with Safi et al. [37]; meanwhile, proteins and carbohydrate contents were opposed to each other [51]. With reference to the best performing ACs (AC_1, AC_10 and AC_12), it seemed that since their µ values were similar, the biochemical composition was the driver in differentiating ACs, i.e., AC_1 was characterized for the highest carbohydrate content, AC_10 for highest protein contents and AC_12 for the highest lipid content (Table 3). 

In general, from the results discussed, it appears that ACs contain less protein and more carbohydrates than pure algae cultures. High levels of biomass carbohydrate contents have been reported as a result of the co-cultivation of algae with bacteria [52]. Choix et al. [53] demonstrated that the co-cultivation of two *Chlorella* strains (*C. vulgaris* and *C. sorokiniana*) with *A. brasilense* raised the positive effect of bacteria on carbohydrate production. In addition, the low N concentration in NFP compared to the N content of synthetic substrates or other wastewaters could explain the low protein content. Generally, algae grown on N-rich wastewater can produce larger quantities of extracellular proteins due to a higher N uptake leading to a high protein content [54].

### 2.6. Amino Acids (AA) and Fatty Acids (FA) Speciation

#### 2.6.1. Amino Acids (AA)

Eighteen amino acids (AA) were identified by analyzing all the biomass produced (Figure 1a). The most abundant amino acids in all ACs were Arg, Asp and Glu, followed by Leu, Gly, Ser, Lys, Pro and Phe (Figure 1a and Table S2). The essential amino acids (EAAs), which include His, Ile, Leu, Lys, Met, Phe, Thr, Trp and Val, were detected in all AC cultures. These data confirmed the high nutritional value of ACs [50].

The most abundant AAs in all ACs reported as average (*n* = 12) values (g 100 g^−1^ crude protein) were as follows: non EAAs: Arg (20.7 ± 1.9) > Asp > (18.6 ± 3.7) > Glu (16 ± 1.4) > Gly (6.9 ± 1.4) = Ser (5.3 ± 0.3) = Pro (4.6 ± 2.3) and EAAS: Leu (6.7 ± 0.6) = Lys (4.9 ± 1.6) = Phe (3.7 ± 0.7) as EAAs. These data were mostly in line with the data reported for *Chlorella* sp. (g 100 g^−1^ crude protein) (*n* = 5), i.e., Arg (7.2 ± 0.9), Asp (9.7 ± 0.6), Glu (11 ± 1.5), Gly (6.3 ± 0.9), Ser (5.5 ± 1.3), Pro (4.6 ± 1.2), Cys (0.9 ± 0.7), Tyr (4.7 ± 1.7), Ala (8.6 ± 0.8), as NEAAs; Leu (8.2 ± 1), Lys (7.1 ± 1), Phe (5.2 ± 0.5), Met (1.4 ± 0.5), Trp (1.8 ± 0.9), Ile (3.6 ± 1.7), His (2.5 ± 1.3), Thr (5.2 ± 0.3) and Val (5.5 ± 1.3) as EAAs [37,48,55,56,57]. The higher Arg, Asp and Glu contents found in this work might be the reason for lower EAA contents, i.e., 24.3 ± 2.8 g 100 g^−1^ crude protein: this value is comparable with average contents of EAAs in *Chlorella* sp. of 36.2 ± 3.7 g 100 g^−1^ crude protein (*n* = 5). The lower EAA contents for the ACs was probably due to the low N content of the substrate (NFP) leading to low protein content, and as a consequence, to lower EAA contents—this latter might be affected negatively because the synthesis of EAAs substantially exhibited slower accumulation compared to that of non-EAAs [58]. In addition, it has been reported for algae that when the N source is mainly represented by ammonium, algal cells quickly assimilate the NH_4_^+^, avoiding toxicity, leading to a net synthesis of Glu [59]. Arginine, as well, has been reported to be preferentially produced by algae when ammonium is the N source [60].

**Figure 2:**
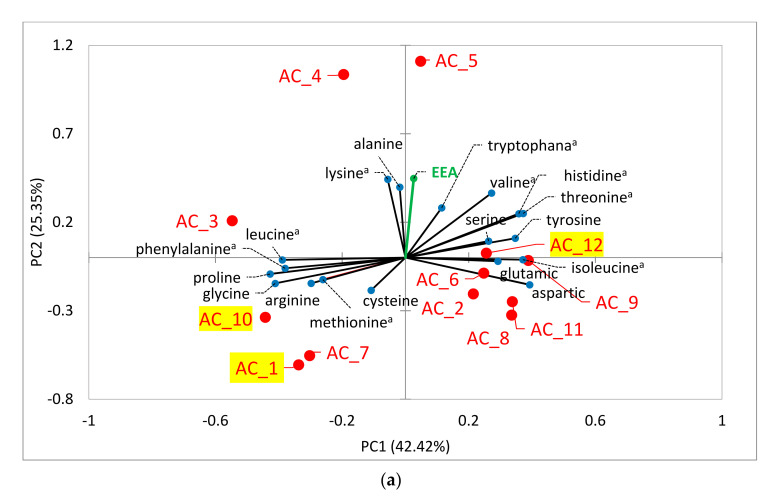

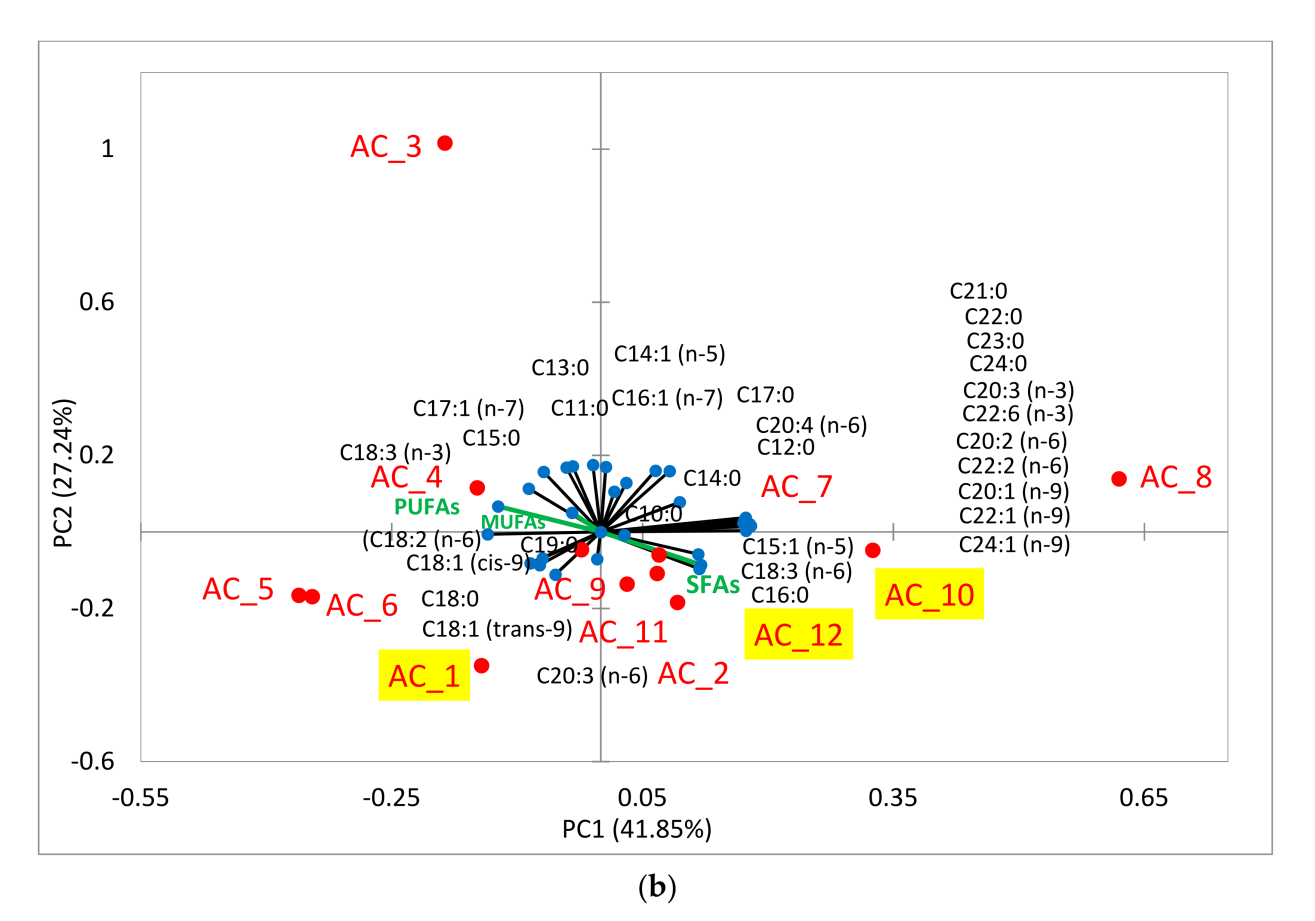
(**a**) Principal component plots for microalgae-microbial consortia (AC) vs. amino acids and (**b**) vs. fatty acids.

Knowledge of AA composition (with particular reference to EAAs) has a great importance in establishing the nutritional value of algae-microbial consortia [61]. Thus, in order to evaluate AA speciation vs. ACs, PCA was performed (Figure 2a). Two main factors (PCs), which accounted for 67.77% of the total variance, i.e., PC1 42.42% and PC2 25.35% of the total variance, were determined (Figure 2a). Generally, most ACs were located in the lower section of PC2, indicating low EAA content amounts, except for AC_4 and AC_5. Notably, AC_5, with the most abundant EAA (30.3 g 100 g^−1^ of crude protein), also showed a quite high protein content (422 ± 3 g kg^−1^ DM), which makes these algae consortia of interest for further application, apart from its low growth rate (µ = 0.31 ± 0.12 d^−1^). 

With reference to the best performing ACs, it can be seen that AC_1 showed an AA composition similar to that of AC_10, in particular for Pro, Gly and Met contents (Figure 2a) that were more abundant than those of AC_12. However, AC_12 showed significantly more abundant EAAs, namely His, Thr, Val and Ile. AC_12 also displayed the second most abundant Trp content, i.e., 50% more than that reported for AC_10, while Trp was not found in AC_1 (Figure 1a). Trp has often been found to be the limiting EAA when assessing the nutritional value of algal proteins [61] in most algae species.

In any case, the three best performing ACs showed relatively low EAA content, representing 20.9 (AC_1), 22.5 (AC_10) and 25.1 (AC_12) g 100 g^−1^ of crude protein, and even for AC_1 and AC_10 EAA contents were lower than the AC average, confirming that higher growth limits protein and EAA contents. Figure 2a suggests that the best performing ACs showed lower EAA contents than those ACs characterized by lower growth rates, especially AC_3, AC_4 and AC_5 (Table S2). Therefore, the least performing ACs (e.g., AC_3, AC_4 and AC_5), due to the high EAA contents and excellent EAA profiles, could be considered healthy and functional for foods. 

Proteins are composed of different amino acids, and hence, the nutritional quality of a protein is basically determined by the content, proportion and availability of its amino acids. The amino acid profiles of the ACs are shown in Table S2 and compared with some basic conventional food items, commercially-used *Chlorella vulgaris* [48] and a reference pattern of a well-balanced protein recommended by WHO/FAO [62]. Among the best performing ACs, AC_12 gained the most favorable amino acids pattern, especially for what concerns Try, Glu, Gly, His, Leu, Lys, Ser, Tyr, Thr and Phe, comparable to that of the reference and other food proteins. Therefore, AC_12 can be proposed as a nutritious protein for further application.

#### 2.6.2. Fatty Acids (FA)

In this work, thirty-two different fatty acids, grouped into saturated fatty acids (SFAs), monounsaturated fatty acids (MUFAs) and polyunsaturated fatty acids (PUFAs), were identified in all ACs (Figure 1b,c). SFAs, PUFAs and MUFAs were, on average, (*n* = 12) 41.2 ± 10.6%, 37 ± 8.6% and 21.9 ± 6.6% of the total lipid content (Figure 1c). These figures are in line with data reported for *Chlorella* sp., i.e., SFAs of 38.1 ± 12.5%, PUFAs of 39 ± 12.9% and MUFAs of 22.8 ± 6.3% of total lipids (*n* = 12), respectively [17,47,63,64], as well as in agreement with the results reported for *Tetradesmus* sp., SFAs 45.2 ± 3.3%, PUFAs 39 ± 15.3% and MUFAs 10.5 ± 6.6% of total lipids (*n* = 4) [47,65,66].

The major SFAs were palmitic acid (C16:0) (37.4 ± 10.4% of total lipids), which was greater than stearic acid (C18:0) (5.2 ± 4.9% of total lipids); whereas linoleic acid (C18:2) (22.2 ± 7.1% of total lipids) was dominant among the unsaturated fatty acids, followed by alpha-linolenic acid (C18:3) (9.4 ± 7.9% of total lipids) which was greater than oleic acid (C18:1 cis-9) (7.8 ± 1.9% of total lipids) and equal to elaidic acid (C18:1 trans-9) (7.8 ± 3.0% of total lipids), on average (*n* = 12) (Figure 1b). These figures agreed with the literature that reported C16:0 as being the most abundant in microalgal consortia [66] as well as in *Chlorella* sp. [67]. Mahapatra et al. [68] reported that the microalgae consortia collected from municipal wastewater contain major contributions from SFAs: C16:0 (42.3% of total lipids) > C18:0 (25.7% of total lipids) > C18:1 (10.9% of total lipids) > C18:2 (around 5% of total lipids). Comparing the data of this work with the literature, it appears that less C18:0, but more unsaturated fatty acids, such as C18:2 and C18:3, were found in ACs’ biomass. Among the three best-performing cultures (AC_1, AC_10 and AC_12), C16:0 was the most abundant saturated fatty acid, with an average content of 47.3 ± 3.2% of total lipids (*n* = 3), which was more similar to the value reported by Mahapatra et al. [68] than the average content of all the ACs (*n* = 12).

In order to evaluate the FA speciation vs. ACs, PCA was performed (Figure 2b). PC1 and PC2 explained 69.09% of the total variance, i.e., 41.85% and 27.24%, respectively. SFAs, MUFAs and PUFAs displayed significant differences between ACs. Higher SFAs led to fewer PUFAs and MUFAs. However, MUFAs did not show much variance between ACs and FAs as they were located near the center. The major PUFAs detected in all cultures were C18 and C20, as well as C22 and C24, but in smaller amounts. In fact, C16 and C18 account for 96.2 ± 2.5% of the total lipids (*n* = 12) that were found in this work. Regarding the three best performing ACs, i.e., AC_1, AC_10 and AC_12, they showed a positive relation with C16:0 and C18:1 (Figure 2b), which are more interesting for the production of biofuel [69]. Biodiesel is a mixture of fatty acid esters consisting mostly of 16 to 18 carbon atoms. The number of carbon atoms present in fatty acid, and their type (SFAs, MUFAs, and PUFAs), are the factors that primarily control the biodiesel properties. A high percentage of any single type of fatty acid (i.e., SFA, MUFA, or PUFA) is not desirable. Ideally, biodiesel must contain a correct mixture of both -SFA (C16) and short-chain MUFA or PUFA (C18) [70]. It can be seen from Figure 2b that the ACs in this work gained various fatty acids in terms of C16 to C18 and quite well-balanced fatty acid types (Figure 1c), suggesting that these best-performing microalgae consortia are suitable for producing biodiesel. 

With reference to the nutritional aspect, the ω6:ω3 (unsaturated fatty acids) ratio becomes important, with the optimum being in the range 4:1-1:1, and ratios above 10:1 promoting pathogenesis of many diseases [71]. The ω6:ω3 ratio of AC_1 and AC_12 were 3.1 and of 3.6, respectively, markedly lower than that of a typical Western diet (16 for USA, 15 for UK and Northern Europe [72]), which demonstrated that these ACs are potential candidates to improve diets by lowering the ω6:ω3 ratio to prevent chronic diseases. Thus, AC_1 and AC_12 can be proposed to be used as feed ingredients, especially for aquaculture and food additives [73], and as a source of omega-3 supplements. Again, from Figure 2b, it can be seen that AC_10 and AC_12 showed a more abundant presence of long-chain FAs (i.e., C20-C24), which have high nutritional value (above all long-chain polyunsaturated fatty acids). 

In conclusion, the three best-performing ACs are recommended to be developed for biofuel production. In addition, among these three, AC_12 (as well as AC 2, AC 7 and AC 11) (Figure 2b) presented high nutritional value because of both the optimal ω6:ω3 ratio and the presence of long-chain polyunsaturated fatty acids. 

Although most of the microalgae consortia have been found to have good nutritional properties because of AA and FA profiles, this is not sufficient to get a final indication because many other factors contribute to the nutritional value of microalga biomass [74]. As a consequence, additional analyses and investigation are required to properly address the suitability of these ACs for practical uses, such as feed and food supplements rather than feed ingredients or plant biostimulants. 

### 2.7. Economic Benefits

Among the best performing ACs, AC_1 and, above all, AC_12 were suggested as potential candidates for nutritional applications, such as animal feed. These ACs showed abilities for taking up nitrogen and phosphorus, indicating that NFP represents a good substrate for them and that isolated microalgae-microbial consortia are well adapted to growing on this waste-derived substrate, producing useful biomasses. Nutrient recovery and biomass production represent an example of the circular economy to be implemented at a local level by using adapted microbial populations. The European market of microalgae is of approximately EUR 918 × 10^6^ y^−1^ [75], and *Chlorella* sp. and *Spirulina* value are of EUR 25–50 kg^−1^ and EUR 30–70 kg^−1^, respectively. Reducing the cost production can be an interesting solution to increasing revenue. Chemical fertilizers represent approximately 7% of total cost, so the use of recovered nutrients (NFP) creates an enticing reduction in total cost [75].

## 3. Materials and Methods

### 3.1. Nanofiltered Permeate Sampling and Characterization

The culture medium used during batch trials was the nano-filtered permeate (NFP) sampled at a full-scale pig slurry treatment plant located in Brescia province in Northern Italy. This plant is a containerized system and operates in automatic and continuous modes, requiring less than 200 m^2^ for its installation. It can treat any kind of livestock manure in a continuous process covering a large range of conditions with a maximum raw input of 120 m^3^ day^−1^. Briefly, the raw slurry was separated after screw-pressing it into a liquid (L1) and solid fraction (S1); then the liquid fraction went through a vibrating screen (0.1–0.3 mm), to give a second liquid (L2) and solid fraction (S2). Lastly, the L2 passed through a 0.01 μm nano-filtration step, thus giving the NFP. In the full-scale plant, NFP is then subjected to a reverse osmosis step, producing a concentrated ammonia solution to be used in agriculture as fertilizer (33% input) and clean water (48% input), that is discharged directly to surface water bodies. Closing the balance, the solid fraction (S1 + S2) accounted for 19% of the input. The liquid fraction contains, on average, 2.3% TS and 3000 mg kg^−1^ TKN, of which more than 80% is ammonium. 

NFP was immediately stored at 4 °C and characterized upon arrival after sampling. Total nitrogen (TN), ammonia nitrogen (NH_4_^+^-N), pH and chemical oxygen demand (COD) were determined on fresh materials according to the analytical methods for wastewater sludge. Macro and microelement contents, including Na, Mg, K, Ca, P, Mn, Fe, Cu, Zn, Cr, Co, Ni, As, Se, Mo, Cd, Pb, were determined by Inductively Coupled Plasma-Mass Spectrometry (ICP-MS, Aurora M90 BRUKER, Bremen, Germany), preceded by microwave-assisted (Multiwave ECO, Anton Paar GmbH, Ostfildern, Germany) nitric acid digestion of fresh samples. All analyses were performed in triplicate.

### 3.2. Microalgae Consortia and Preparation of Inoculum

The algae consortia used in these trials were obtained previously by direct isolation from organic wastes [20] sampled directly at full-scale plants located in Northern Italy, aiming to select algae-microbial consortia well adapted to substrates rich in C and high in nutrient contents. All collected samples were immediately brought to the laboratory and stored at 4 °C before further analyses. Preliminary experiments were performed in order to assess the best conditions able to isolate the greatest number of algae [8]. In brief, the sixteen organic waste samples were used as the sources for isolating native microalgae-microbial consortia. Four groups of preliminary experiments (1–4) were performed. Preliminary experiment 1 and 2 used deionized water and BG-11 nutrient solution for enrichment and isolation. Generally, waste (2g) and 200 mL deionized water/BG-11 were dispensed into 250 mL sterilized Erlenmeyer flasks. The mixture was mixed with medium and agitated manually for 10 min before putting into the incubator. Optical densities were not unified. This approach gave low results in terms of isolated consortia [8]. 

Experiment 3 and 4 considered the use of three widely-used algae cultivating nutrition media, namely: CA medium, Bold’s Basal Medium (BBM) and BG-11 medium. Optical density was uniform at 0.1 and 0.3. The flasks were then incubated for enrichment under constant aeration and mixed by using filtered air (filter of 0.2 µm) with a continuous illumination of 50 µE m^−2^ s^−1^, provided by fluorescent white tubes, at a controlled temperature of 22 ± 1 °C for 8 weeks. BG 11 and an optical density of 0.3, resulted in the best substrate isolating ACs that were then used [8].

Isolated algae consortia (AC) (Table 2) were maintained in 500 mL Erlenmeyer flasks in BG-11 medium under constant aeration and mixed by using filtered air (filter of 0.2 µm) with a continuous illumination of 50 µE m^−2^ s^−1^, provided by fluorescent white tubes, at a controlled temperature of 22 ± 1 °C. Afterwards, the twelve algae consortia isolated from the sixteen organic wastes were subject to metagenomic characterization by Next Generation Sequencing (NGS) analysis [8].

### 3.3. Microalgae Consortia Molecular Characterization

DNA extraction was carried out during the exponential growth phase. The consortia biomasses were collected by centrifugation at 4000 rpm for 10 min and 8000 rpm for another 10 min and then stored at −80 °C until further analysis. Then the lyophilized biomass samples were collected for DNA extraction by DNeasy plant mini kit Qiagen, following the procedure described by the manufacturer. The extracted DNA samples were stored at −20 °C for further use. DNA concentration and purity were determined by a nanodrop 1000 spectrophotometer (Thermo Fisher Scientific Inc., Pleasanton, CA, USA).

For NGS, a library for 16S and 18S marker genes was prepared following Illumina Protocol. For the 16S, the hypervariable V3–V4 region was amplified using the 341F and 805R primers while for 18S, the V9 region was amplified using the 1389F and 1510R primers both modified with the required Illumina sequencing adaptors. PCR products were quantified using PicoGreen^®^ dsDNA quantification assays (Thermo Fisher Scientific), on a POLAR star Omega (BMG Labtech) plate reader. Nextera XT amplicons were then pooled in equimolar concentration. The length of amplicons was verified with an Agilent bioanalyzer DNA kit (Agilent, Santa Clara, CA, USA). Final quantification of the pooled amplicon library was determined with the NEBNext^®^ Library Quant Kit for Illumina^®^ (New England BioLabs, Ipswich 01938, MA, USA) prior to sequencing on the Illumina MiSeq (2 × 300 bp) at the University of Essex (Colchester, UK). 

### 3.4. Experimental Set-Up and Cultivation

This study aimed to obtain the first data about growth performance and chemical and biological characteristics of algae consortia previously isolated from organic wastes, using a waste stream, i.e., pig slurry derived product (NFP), as culture medium (see Section 3.1). 

To do so, batch trials were carried out in triplicate in self-built photo-bioreactors (PBRs), composed of glass vessels of 0.5 L working volume, supplied with air through plastic pipes connected to an air compressor equipped with a flowmeter and supplied with CO_2_ through a CO_2_ cylinder. pH was set at 8 and was maintained by using a pure CO_2_ injection adopting an “on-demand” modality [76]. Room temperature (25 °C) and constant air flux (10 L min^−1^) were provided as well as light that was delivered by cold fluorescent lamps at an irradiance of 312 μE m^−2^ s^−1^ at the PBR surface. A 12 h:12 h photoperiod regime was selected as already proposed in previous works for lab culturing of both pure microalgae and microalgae consortia by providing the light regime suitable for almost all strains present in the inocula [77,78]. NFP was used as the batch growth medium; P (7.11 mg L^−1^ of K_2_HPO_4_) and Fe (1.02 mg L^−1^ (NH_4_)_5_[Fe(C_6_H_4_O_7_)_2_]) were added, to provide a complete growth substrate.

### 3.5. Microalgae Growth Determination

Microalgae dry weight (DW) was determined by sampling 5 mL of algae suspension from each PBR. The samples were centrifuged at 4000 rpm for 10 min and then washed with an equivalent volume of distilled water to remove salts. Culture samples were then filtered by 1.2 µm filters (GF/C, Whatman Ltd., Maidstone, UK), dried overnight at 80 °C and weighed. Sampling was done every day. 

The specific growth rate *µ* (day^−1^) was calculated from Equation (1):

*µ* = (*lnN*_1_ − *lnN*_2_)/(*t*_1_ − *t*_2_) (1)

where *N*_1_ and *N*_2_ are the concentrations of cells (g L^−1^) at day *t*_1_ and *t*_2_.

The Nitrogen (*N*) taken up by biomass was calculated according to Equation (2):

*N taken up by biomass* = (*TN_biomass_* × *DB*)/(*N_initial_*)% (2)

in which *TN_initial_* (mg L^−1^) is the nitrogen concentration at the beginning, *TN_final_* (mg L^−1^) is the nitrogen concentration at the end of the experiment, *TN_biomass_* (mg kg^−1^) is the concentration of *TN* in the biomass and *DB* (kg L^−1^) is the dry biomass produced per L of growth medium.

The Phosphorus (*P*) taken up by biomass was calculated according to Equation (3) [35]:

*P taken up by biomass* = (*P_initial_* − *P_final_*)/*P_initial_*% (3)

in which *P_initial_* (mg L^−1^) is the phosphorus concentration at the beginning and *P_final_* (mg L^−1^) is the phosphorus concentration at the end of the experiment.

### 3.6. Biochemical Analysis

The total N concentration (*TN_biomass_*, g kg^−1^ DM) was detected on biomass samples of about 0.2–0.3 g, using an elementary analyzer (Elementar Rapid max N exceed, Elementar Italia s.r.l., Lomazzo, Italy), based on the analytical method of combustion “Dumas” and equipped with a thermal conductivity detector (TCD, Elementar Italia s.r.l., Lomazzo, Italy). The crude protein contents were estimated by multiplying the total N by the conversion factor 6.25 [61]. The ash content was determined as the residue after ignition at 550 °C overnight. Carbohydrates were estimated by subtracting the percentage of ashes, lipids and crude proteins out of 100%. The mass balance was verified as the residual fraction (composed of carbohydrates and soluble non-protein cell content), calculated as the missing part to the total weight, as previously proposed [79].

The amino acid (AA) content of algal biomass was determined by the HPLC-DAD technique with some modifications [61]. Acidic hydrolysis of samples was used for the determination of lysine (Lys), histidine (His), phenylalanine (Phe), isoleucine (Ile), leucine (Leu), valine (Val), threonine (Thr), arginine (Arg), alanine (Ala), glycine (Gly), proline (Pro), glutamic acid (Glu), serine (Ser), aspartic acid (Asp) and tyrosine (Tyr). About 0.1–0.2 g of freeze-dried samples were hydrolyzed in 10 mL of 6 Mol L^−1^ HCl for 24 h in a water bath at 100 °C, followed by neutralization with NaOH. For the determination of sulfur amino acids (Met and Cys), the samples were pre-treated with 1 mL of a mixture of 30% (*v*/*v*) hydrogen peroxide and 98% (*v*/*v*) formic acid (in the ratio of 1:9 *v*/*v*) and were subsequently hydrolyzed in the way described above. For tryptophan (Trp) determination, an alkaline hydrolysis was performed: about 0.1–0.2 g of freeze-dried sample was hydrolyzed with 10 mL of NaOH 4.2 Mol L^−1^ for 16 h under N_2_ flux and neutralized with HCl. The HPLC analyzer (Agilent 1100 Series HPLC, Agilent, Santa Clara, CA USA) tests were performed by automated online pre-column derivatization using an automated liquid sampler and Poroshell 120 column HPH-C18 (3.0 100 mm, 2.7 lm. P/N 695975–502). For the standard preparation derivatization process, the LC method used was performed according to Agilent Pub. #5990-4547EN (Pub No. 5990-4547EN, October 8, 2009, Agilent Technologies). The primary amino acids (OPA-derivatized) were monitored at 338 nm. The secondary amino acids (FMOC-derivatized) were monitored at 262 nm. For methionine (Met) and cysteine (Cys) detection, DTDPA (3,3-dithiodipropionic acid) was used as the derivatizing agent instead of FMOC. The separation was carried out under gradient elution with two mobile phases. Phase A: 10 mM NaH_2_PO_4_ + 10 mM Na_2_B_4_O_7_ + 5 mM NaN_3_, pH 8.2 adjusted with HCl 5 M, and Phase B: ACN:MeOH:water (45:45:10, *v*/*v*/*v*). The flow rate was 1.00 mL min^−1^, the column temperature 40 °C and injection volume 20 µL.

Total lipids were determined using a slightly modified version of the Bligh and Dyer [80] method. An aliquot of lyophilized biomass was mixed with 600 μL of chloroform:methanol (2:1 *v*/*v*), after mixing well, 200 μL of methanol and 333 μL of deionized water were added. The mixtures were then transferred into a separator funnel and shaken for 5 min. The lipid fraction was then collected from the separator funnel and gravimetrically determined after evaporation over one night. For fatty acid compositional analysis, the microalgae oils were esterified as suggested by the Sigma Aldrich Fatty Acid Methyl Ester Preparation Protocol and used for GC-MS analysis. Chromatographic analysis was performed using an Agilent 5975C Series GC/MSD and FID as a detector (Agilent, Santa Clara, CA, USA). Volatiles were separated using a polar capillary column Zebron ZB-FAME (Zebron, Phenomenex, Castel Maggiore, Italy) of 30 m x 0.25 mm (ID) and a film thickness of 0.20 µm. The injection volume was 1 µL with a split ratio of 20:1. Carrier gas was helium at a flow rate of 1 mL min^−1^. The temperature program was isothermal for 2 min at 100 °C, then the temperature was raised at a rate of 3 °C min^−1^ to 240 °C and kept at 240 °C for 5 min. Injection temperature was 250 °C and the transfer line to the mass spectrometer was maintained at 285 °C. The mass spectra were obtained by electronic impact at 70 eV, a multiplier voltage of 1294 V and collecting data at an *m*/*z* range of 45–300. Compounds were identified and quantified by comparing their mass spectra and retention times (RT) with those from the standards contained in the Supelco 37 Component FAME Mix provided by Supelco, Sigma Aldrich (Saint Louis, MO, USA). Heptadecane was used as an internal standard.

### 3.7. Data Analysis

Data were processed by one-way ANOVA (analysis of variance), the Tukey’s HSD multiple comparison tests (p < 0.05) to compare means and multivariate analyses, i.e., principal component analysis (PCA), using XLSTAT version 2016.02.28451 (New York, NY, USA).

## 4. Conclusions

Twelve microalgae-microbial consortia isolated from organic wastes were tested for their ability to grow on nanofiltered swine manure (NFP). *Chlorella*-dominated consortia (AC_1), *Tetradesmus* and *Synechocystis* co-dominated consortia (AC_10) and *Chlorella* and *Tetradesmus* co-dominated consortia (AC_12) exhibited the highest specific growth rates. Generally, the best performing ACs showed poor amino acid profiles in terms of essential amino acids along with relatively lower amounts of PUFAs in fatty acid profiles than the rest of the ACs. However, they can be potentially applied for biofuel production and food supplements (above all the AC_12). Moreover, NFP could address the specific issue of organic cultivation of microalgae for food where, at present, chemical fertilizers are not allowed. For organic culturing of microalgae, companies in Europe use vegetable organic fertilizers, containing organic nitrogen that is quite inefficient for microalgae, and which cost accounts for EUR 2–3 kg^−1^ of microalgae. The use of NFP could decrease the cost of nitrogen supply to EUR 0.15–0.20 kg^−1^ of microalgae. 

The results obtained in this work provided preliminary information on the ACs’ performances because the monitoring of the microbial population and community composition in each stage of cultivation has been neglected. More exhaustive and reliable data about physiological performance, productivities, biochemical composition and consortia evolution during the cultivation will be obtained by experimental trials that will be conducted in continuous culture in an open pond at pilot scale, providing practical information for large-scale applications.

## Figures and Tables

**Table 1:** NFP chemical characterization vs. BG-11 nutrient solution.

		NFP	BG-11
pH		8.5 ± 0	7.4
TN	mg L^−1^	136 ± 0	247
NH_4_-N	mg L^−1^	132 ± 2	19
COD	mg L^−1^ O^2^	77 ± 4	-
P	mg L^−1^	7.61 ^a^	7.11
Na	mg L^−1^	249 ± 3	414
Mg	mg L^−1^	5.4 ± 0.1	7.39
K	mg L^−1^	188 ± 8	17.95
Ca	mg L^−1^	9.7 ± 0.2	9.81
Fe	mg L^−1^	1.42 ^b^	1.42
B	mg L^−1^	0.5 ± 0.1	0.50
Al	mg L^−1^	0.6 ± 0.0	n.p. ^e^
Cr	μg L^−1^	4.7 ± 0.6	n.p.
Co	μg L^−1^	4.8 ± 1.2	10
Cu	μg L^−1^	30.7 ± 0.4	30
Zn	μg L^−1^	57 ± 16	50
Se	μg L^−1^	5.2 ± 0.4	n.p.
Mo	μg L^−1^	19 ± 5	150
Cd	μg L^−1^	7 ^c^	n.p.
Pb	μg L^−1^	6.5 ± 2	n.p.
As	μg L^−1^	u.d.l ^d^	n.p.
Mn	μg L^−1^	u.d.l	500
Ni	μg L^−1^	u.d.l	n.p.

^a^ P content in NFP was 0.5 ± 0, p was added, getting a final content of 7.61 mg L^−1^. ^b^ Fe content in NFP was 0, Fe was added, getting a final content of 1.42 mg L^−1^. ^c^ the other replicates are under detection level. ^d^ u.d.l refers to under detection level. ^e^ not present.

**Table 2:** Algae consortia main genus composition and growing performance.

	Eukaryotic Genus		Prokaryotic Genus		µ
	Algae % ^a,f^	Other Eukaryotes % ^a,f^	Algae % ^b,g^	Other Prokaryotes % ^b,g^	d^−1^
AC_1	*Chlorella* 99.1%	n.f. ^c^	n.f.	*Paludisphaera* (Planctomycetota) 36%	0.55 ± 0.04a ^e^
AC_2	*Chlorella* 8.4%	*Nuclearia* 40.6%; *Vahlkampfia* 30.7%; *Colpoda* 15.6%	*Synechocystis* 35.9%	*Truepera* (Deinococcata) 21%	0.22 ± 0.03b
AC_3	*Chlorella* 85%	-	*Synechocystis* 19.6%	*SM1A02* (Planctomycetota) 36.8%	0.25 ± 0.04b
AC_4	*Chlorella* 76.4%	*Colpoda* 10.3%	*Synechocystis* 27.9%	*SM1A02* (Planctomycetota) 34.5%	0.31 ± 0.12b
AC_5	*Chlorella* 30.6%	*Colpoda* 36.1%; *Nuclearia* 17.7%	*Synechocystis* 84.8%	n.f.	0.29 ± 0.04b
AC_6	*Tetradesmus* 85.4%	*Colpoda* 9%	n.f.	Others ^d^ 61%	0.31 ± 0.02b
AC_7	*Tetradesmus* 42.6%	*Colpoda* 34.8%	*Synechocystis* 21.4%	*Chloronema* (Chloroflexi) 22.9%	0.24 ± 0.02b
AC_8	*Scenedesmus* 8.1%; *Chlorella* 6.3%	*Colpoda* 69.3%	n.f.	*SM1A02* (Planctomycetota) 42.5%	0.28 ± 0.08b
AC_9	*Chlorella* 82.3%	*Vermamoeba* 11.9%	*Synechocystis* 35.4%	*SM1A02* (Planctomycetota) 34.3%	0.31 ± 0.02b
AC_10	*Tetradesmus* 98.4%	n.f.	*Synechocystis* 54.2%	n.f.	0.52 ± 0.06a
AC_11	*Chlorella* 34.5%	*Cyclidium* 34.1%	*Synechocystis* 9.2%	*SM1A02* (Planctomycetota) 57.7%	0.18 ± 0.01b
AC_12	*Chlorella* 39.6%; *Tetradesmus* 32.6%	*Vermamoeba* 9.4%	*Synechocystis* 3.6%	*Sandaracinus* (Proteobacteria) 29.8%; Others ^c^ 52.5%	0.58 ± 0.06a

^a^ Genus composition in microalgae consortium eukaryotic community. ^b^ Genus composition in microalgae consortium prokaryotic community. ^d^ not found. ^c^ Others refers to an undetectable composition in prokaryotic community. ^e^ Means followed in the same column by the same lower-case letter are not statistically different (*p* < 0.05) according to Tukey test. ^f^ Percentages refers to the relative abundance of OTUs assigned to each genus with respect to total OTUs assigned to Eukaryotes. ^g^ Percentages refers to the relative abundance of OTUs assigned to each genus with respect to total OTUs assigned to Prokaryotes.

**Table 3:** Nitrogen and phosphorus mass balance and biochemical compositions.

AC	TN_initial_^a^	TN_final_^b^	N_biomass_^c^	N Taken Up by Biomass	P_initial_^d^	P_final_^e^	P Taken Up by Biomass	Proteins	Lipids	Carbohydrates
	mg L^−1^	mg L^−1^	g kg^−1^ DM ^f^	% TN_initial_^a^	mg L^−1^	mg L^−1^	% P_initial_^d^	g kg^−1^ DM	g kg^−1^ DM	g kg^−1^ DM
AC_1	136 ± 0	35 ± 6	41 ± 0	54 ± 0	7.61 ± 0	0.49 ± 0.01	94 ± 0	257 ± 0g ^g^	119 ± 1fg	596 ± 4
AC_2	136 ± 0	23 ± 1	74 ± 0.6	65 ± 1	7.61 ± 0	1.13 ± 0.16	85 ± 2	460 ± 4b	105 ± 7g	405 ± 9
AC_3	136 ± 0	12 ± 3	90 ± 0.2	59 ± 0	7.61 ± 0	0.72 ± 0.37	91 ±5	561 ± 1a	152 ± 10cde	254 ± 11
AC_4	136 ± 0	20 ± 4	68 ± 0.4	78 ± 0	7.61 ± 0	0.40 ± 0.14	95 ± 2	422 ± 3c	177 ± 3b	359 ± 6
AC_5	136 ± 0	45 ± 13	43 ± 0.1	76 ± 0	7.61 ± 0	0.47 ± 0.08	94 ± 1	266 ± 0g	153 ± 9bcde	565 ± 9
AC_6	136 ± 0	29 ± 4	49 ± 0.7	65 ± 1	7.61 ± 0	0.45 ± 0.17	94 ± 2	305 ± 5f	173 ± 11bc	486 ± 12
AC_7	136 ± 0	3.4 ± 0.5	53 ± 0.7	46 ± 0	7.61 ± 0	0.54 ± 0.03	93 ± 0	334 ± 4e	128 ± 8efg	512 ± 9
AC_8	136 ± 0	38 ± 2	67 ± 0.4	53 ± 0	7.61 ± 0	0.51 ± 0.11	93 ± 1	420 ± 2c	177 ± 9b	369 ± 10
AC_9	136 ± 0	35 ± 7	64 ± 0.3	87 ± 0	7.61 ± 0	0.36 ± 0.08	95 ± 1	398 ± 2cd	178 ± 8b	406 ± 8
AC_10	136 ± 0	31 ± 1	61 ± 0.1	62 ± 0	7.61 ± 0	0.43 ± 0.08	94 ± 1	382 ± 7d	156 ± 9bcd	425 ± 9
AC_11	136 ± 0	27 ± 5	55 ± 0.3	29 ± 0	7.61 ± 0	1.37 ± 0.37	82 ± 5	341 ± 2e	135 ± 4def	494 ± 5
AC_12	136 ± 0	21 ± 1	44 ± 0.6	53 ± 11	7.61 ± 0	0.46 ± 0.01	94 ± 0	273 ± 22g	230 ± 15a	472 ± 27

^a^ Initial TN concentration of culture medium at the start of the experiments. ^b^ TN concentration of culture medium at the end of the experiments. ^c^ TN concentration in AC biomass. ^d^ Initial P concentration of culture medium at the start of the experiments. ^e^ P concentration of culture medium at the end of the experiments. ^f^ DM refers to dry matter. ^g^ Means followed in the same column by the same lower-case letter are not statistically different (*p* < 0.05) according to Tukey test.

## Data Availability

Data is contained within the article.

## Supplementary Materials

The following supporting information can be downloaded online, Table S1: Organic wastes sampling and origin details. Table S2: Amino acids compositions in ACs.
