# Supplementary material for: Growth Performance, Biochemical Composition and Nutrient Recovery Ability of Twelve Microalgae Consortia Isolated from Various Local Organic Wastes Grown on Nano-Filtered Pig Slurry
Source: Molecules. 2022 Jan 10;27(2):422. doi: 10.3390/molecules27020422 (PMC8781922; doi:10.3390/molecules27020422)
Supplement: Supplementary file 1 [file molecules-27-00422-s001.zip › molecules-1516627-supplementary.pdf]

Supplementary material for:

Growth performance, biochemical composition and nutrients recovery ability of twelve microalgae consortia isolated from various local organic wastes grown on nano-filtered pig slurry

Min Su[1], Marta Dell'Orto[1], Barbara Scaglia[1], Giuliana D'Imporzano[1], Alessia Bani[2] and Fabrizio Adani[1,]*

Table S1. Organic wastes sampling and origin details

	Sample Name	Origin	Storge Mode	Digestion Type	Location	Plant/farm Scale	Plant Volume m^3	Sampling Temperature ℃	Ambient Temperature ℃
S1	Cow slurry	Cattle Stables	Open lagoon	-	Mantova	150 animals	-	8	4
S2	Liquid digestate of cow slurry	Biogas Plant	Open storage	Mesophilic	Suzzara	1 MW, 5 digesters	12000	15	3.5
S3	Solid digestate of cow slurry	Biogas plant	Open storage	Mesophilic	Suzzara	1 MW, 5 digesters	12000	10	3.5
S4	Liquid digestate of cow slurry	Biogas plant	Open storage	Mesophilic	Pegognaga	1 MW, 4 digesters	10000	20	3
S5	Cow slurry	Cattle Stables	Open lagoon	-	Pegognaga	100 animals	-	9	3
S6	Solid digestate of cow slurry	Biogas plant	Open storage	Mesophilic	Pegognaga	1 MW, 4 digesters	10000	10	3
S7	Cow slurry	Cattle stables	Open lagoon	-	Lodi	400 animals	-	10	2
S8	Digestate of cow slurry	Cattle stables	Open storage	Mesophilic	Bologna	-	-	15	2
S9	Wastewater	Urban wastewater treatment plant	Open lagoon	-	Peschiera Borromeo	$5.2x10^5$ equivalent inhabitants	154095 m^2	10	4
S10	Digestate of OFMSW[a]	Biogas plant fed by OFMSW	Open storage	Thermophilic	Lodi	1 MW, 4 digesters	8000	20	7
S11	Liquid fraction of OFMSW	Biogas plant fed by OFMSW	Closed lagoon	-	Lodi	1 MW, 4 digesters	8000	10	7
S12	OFMSW	Biogas plant fed by OFMSW	Closed lagoon	-	Lodi	1 MW, 4 digesters	8000	10	7
S13	Sewage Sludge	Biogas Plant	Open lagoon	-	Vellezzo Bellini (PV)	1.6 MW, 3 digesters	13500	15	8
S14	Digestate of sewage sludge	Biogas Plant	Closed storage	Thermophilic	Vellezzo Bellini (PV)	1.6 MW, 3 digesters	13500	20	8
S15	Cow slurry	Cattle stables	Open storage	-	Bologna	-	-	8	2
S16	Cattle manure	Cattle stables	Open storage	-	Lodi	-	-	10	5

[a]OFMSW: Organic fraction of municipal solid wastes

Table S2. Amino acids compositions in ACs.

	AC_1	AC_2	AC_3	AC_4	AC_5	AC_6	AC_7	AC_8	AC_9	AC_10	AC_11	AC_12	WHO/FAO [63]	Egg [62]	Soybean [62]	*Chlorella vulgaris* [62]
	AA content (g 100 g^{-1} protein)															
cysteine	4.2	0.6	0.3	0.4	0.3	0.7	0.6	0.5	0.5	0.3	0.4	0.7		2.3	1.9	1.4
methionine[a]	2	1.2	1.7	1.2	0	1.2	0.6	0.4	0.5	0.9	0.7	0.4	3.5	3.2	1.3	2.2
tryptophana[a]	0	0.4	1.6	1.3	1.9	1.3	0	0.5	2.3	1	0.6	2	1.0	1.7	1.4	2.1
aspartic	14.5	19.1	14.1	15.3	15	21.1	17.2	22.7	23.9	16.5	25.2	19.3	-	11.0	1.3	9.0
glutamic	15.7	16.8	12.8	14.1	18.3	15.4	15.8	17.1	17.3	16.2	16.4	16.3	-	12.6	19.0	11.6
alanine	0	0	0	0.4	0.9	0	0	0	0	0	0	0	-	-	5.0	7.9
arginine	21.2	21.9	21.9	19.2	20.3	20.2	23.7	20.1	17.2	23.1	17.6	21.5	-	6.2	7.4	6.4
glycine	7.5	5.9	9.6	6.4	5.7	6.3	9.1	6	5.7	9.2	5.9	5.8	-	4.2	4.5	5.8
isoleucine[a]	1.3	1.6	1.2	1.2	1.5	1.8	1.2	1.5	1.5	1.3	1.5	1.9	4.0	6.6	5.3	3.8
histidine[a]	0.3	1.3	0	1.6	1.4	1.3	0	1.1	1.4	0.2	1.2	1.1	-	2.6	2.4	2.0
leucine[a]	6.9	6.9	7.4	7.4	6	6.1	7	6	6	7.6	6.3	6.4	7.0	8.8	7.7	8.8
lysine[a]	4.1	3.7	5.6	7.9	8.5	4.3	3.8	4	4.7	3.4	4.2	4.1	5.5	5.3	6.4	8.4
proline	9.4	3.2	7.6	5.5	3.4	2.8	6.2	2.7	2.5	6.8	2.7	2.8	-	4.2	5.3	4.8
serine	4.8	5.9	5.3	5.5	5.1	5.5	5	5.6	5.2	5	5.4	5.5	-	6.9	5.8	4.1
tyrosine	1.9	2.9	2.4	2.9	2.5	3	2.3	3	3.2	0.6	3.1	3	-	4.2	3.7	3.4
threonine[a]	1.3	3.3	1.8	3.5	3.4	3.3	1.3	3.2	3.1	1.3	3.2	3.2		5.0	4.0	4.8
valine[a]	1.7	2.2	1.9	2.5	2.7	2.2	1.7	2.2	2.2	1.8	2.2	2.4	5.0	7.2	5.3	5.5
phenylalanine[a]	3.4	3.2	4.8	3.7	3.2	3.5	4.5	3.3	2.8	4.9	3.3	3.7	6.0	5.8	5.0	5.5
EEA %	20.9	23.8	26	30.3	28.5	25	20.1	22.2	24.5	22.5	23.2	25.1	-	-	-	

[a] Essential amino acids (EAAs)
